# Predictors of sickness absence in college and university educated self-employed: a historic register study

**DOI:** 10.1186/1471-2458-14-420

**Published:** 2014-05-02

**Authors:** Liesbeth EC Wijnvoord, Jac JL Van der Klink, Michiel R De Boer, Sandra Brouwer

**Affiliations:** 1 Department of Health Sciences, Community and Occupational Medicine, University Medical Center Groningen, University of Groningen, 1, 9713 AV, Groningen, The Netherlands; 2 Movir, PO Box 21603430 CV Nieuwegein, The Netherlands; 3 Department of Health Sciences and the EMGO Institute for Health and Care Research, Faculty of Earth and Life Sciences, VU University, Amsterdam, The Netherlands

**Keywords:** Disability insurance, Risk factors, Sick leave, Absenteeism, Self-employed, Predictive variables

## Abstract

**Background:**

Despite a large proportion of the workforce being self-employed, few studies have been conducted on risk factors for sickness absence in this population. The aim of this study is to identify risk factors for future sickness absence in a population of college and university educated self-employed.

**Methods:**

In a historic register study based on insurance company files risk factors were identified by means of logistic regression analysis. Data collected at application for private disability insurance from 634 applicants were related to subsequent sickness absence periods of 30 days or more during a follow-up period of 7.95 years. Variables studied were self-reported lifestyle variables, variables concerning medical history and present health conditions and variables derived from the general medical examination including blood tests and urinary analysis.

**Results:**

Results from analysis of data from 634 applicants for private disability insurance show that previous periods of sickness absence (OR 2.07), female gender (OR 2.04), health complaints listed in the health declaration (OR 1.88), elevated erythrocyte sedimentation rate (ESR) (OR 4.05) and the nature of the profession were related to a higher risk of sickness absence.

**Conclusions:**

Sickness absence was found to be related to demographic variables (gender, profession), medical variables (health complaints and erythrocyte sedimentation rate) and to variables with both a medical and a behavioural component (previous sickness absence).

## Background

In 2012 there were over 30 million self-employed in the European Union and nearly 15 million in the USA [1,2]. Despite this being a large proportion of the workforce, few studies have focussed on predictors of sickness absence and disability in this group. Most studies have been conducted in employed populations, i.e. individuals working for an employer. It is uncertain whether the findings regarding predictors for sickness absence in employees can be fully applied to the self-employed since the two populations have altogether different profiles, systems of payment and working conditions. Those in self-employment are described as being in better health [3] less often absent from work in comparison to employees [4] and are more satisfied with their work [5,6], to have higher work engagement [7] and different coping strategies [8]. On the other hand, self-employment may not always be a positive choice. Lack of other attractive employment possibilities can force individuals into self-employment [9]. The risks, insecurities and workload associated with being self-employed may cause increased levels of stress [10].

At the application stage for insurance, variables thought to be predictors of sickness absence are used by insurers to estimate the risk of having to pay insurance claims. Accurate risk assessment is important as the basic principle, that underlies all insurance products, is that the premium paid is proportional to the risk of future claims (actuarial fairness). Correct classification of risks is important not only for the insurance company but also for the insured as too many high-risk individuals in a risk pool may force the insurer to increase the premiums thus affecting all policyholders. Another important aspect is that sickness absence and long-term disability present considerable problems for the self-employed because, in the absence of colleagues to take over work, it impacts on the continuity of business and often leads to loss of personal income. Therefore, it is also important to identify risk factors for future periods of sickness absence in self-employed to recognise vulnerable groups and to develop strategies to prevent health problems and sickness absence.

In 2006, Bakker et al. performed a literature review to identify which risk factors for disability in self-employed were described [11]. From eight studies only two studies addressing predisposing risk factors, predicting the onset of sickness absence in self-employed individuals were found [12,13]. The other studies focussed on perpetuating factors, influencing the duration of absenteeism [11]. It was concluded that relevant predisposing risk factors for the self-employed were demographic factors (gender, age, occupational class and socio- economic status), medical and behavioural factors (medical consumption, lifestyle, coping behaviour and previous sick leave) and insurance-related factors (replacement ratio and policy terms).

Since then, no additional studies have been conducted on predisposing risk factors for disability or sickness absence in healthy self-employed workers. Knowledge about predisposing risk factors for sickness absence in self-employed is therefore scarce. The aim of the present study was therefore to identify predictors of future sickness absence in a population of self-employed and to evaluate risk assessment procedures at the application stage used by insurers (Additional file 1).

## Methods

### Sample and study design

In the Netherlands self-employed who wish protection against the financial risks of disability are not covered by public disability insurance systems, but have to apply for private disability insurance. Insurance company files can therefore provide knowledge concerning sickness absence and disability in this group. We used historic data from applicants for a private disability insurance policy at a company insuring only college and university educated self-employed, e.g. doctors, lawyers or dentists. At the application stage the insurance company collected medical and non-medical data that were thought to be suitable for risk assessment. This data was combined with data on subsequent periods of sickness absence. Included were all applicants who applied for an insurance policy with a deferment period, i.e. a waiting period before the insurance company starts paying benefits of 30 days, underwent a general medical examination consisting of a physical examination, blood tests and urinary analysis, were accepted for insurance cover in 2003 and still had their insurance policy by July 1, 2011. The follow-up period started the day the applicant was accepted for disability insurance cover.

Ethical approval was sought from the Medical Ethics Committee of the University Medical Centre Groningen, which advised that, according to Dutch law, ethical clearance was not required for this study.

### Procedures and measurements

At the application stage all applicants completed a health declaration form. If the sum insured was over 50 euros per day, a standard medical examination took place conducted by general practitioners or specialised institutes using a standard examination form. The decision to have a standard examination performed was not related to health characteristics of the applicant, only to the amount insured. Various certified laboratories were used for analyses of blood and urine samples. In 56 applicants an examination was performed for another purpose (e.g. life insurance) shortly before insurance application and these reports, although not always containing complete data, were used by the insurance company for risk assessment.

The dependent variable in this study was sickness absence. Since the shortest possible deferment period for the insurance company studied is 30 days, only periods of sickness absence of 30 days or more were included, as these were reliably administrated by the insurance company. Sickness absence vs. no sickness absence was chosen as this corresponds best with the way insurance companies assess risks, in which the occurrence of sickness absence periods is more relevant than the time to this event. No distinction was made between partial and total disability. Duration of sickness absence was thus defined as the number of days a claim was paid by the insurance company because of inability of the insured person to perform his or her own work fully. The inability to work was judged by the insurance company physician using medical information from treating physicians and data from self-report. Included was sickness absence due to both somatic and mental causes. Although the private insurance company studied provides a benefit for normal pregnancies, these were not included in the case definition. However, pregnancy related disability caused by complicated pregnancies was included.

The potential predictors consisted of all variables that were assessed in the health declaration, the medical examination and age at application, gender and profession. In the former, applicants were requested to provide details concerning their smoking status (present/former/never and number of cigars or cigarettes; operationalized as current smoker yes/no) and the number of alcohol consumptions a day (operationalized as ≤2 versus > 2 consumptions a day) and to answer questions regarding their lifetime medical history such as consultation of health professionals (GP yes/no, medical specialists yes/no, physiotherapist yes/no, psychologist or social worker yes/no, other health professional yes/no) operations or accidents, present health problems, life style (sports yes/no) and work (over or under 50 hours worked/week). Questions regarding the family history of cardiovascular and psychiatric problems were asked as well (yes/no). The standard medical examination consisted of a physical examination (including length in cm, weight in kg and waist and hip circumference in cm, pulse, and blood pressure in mm Hg) electrocardiogram, urine tests and blood tests. Blood tests generally included haemoglobin, erythrocyte sedimentation rate (ESR), fasting blood glucose, liver enzymes (most often gamma-glutamyl transferase (GGT), sometimes other liver enzymes), lipids (most often cholesterol and HDL-cholesterol, sometimes LDL-cholesterol and triglycerides). Urine tests included qualitative testing for protein and glucose (dipstick) and often a sediment. For measurement units of blood tests used see Table 1. Characteristics of the insurance contract such as the replacement ratio were not studied because insured persons tend to change the amount of insured daily compensation over the years, hence the sum originally insured would not be an accurate representation of the replacement ratio at the time of sickness absence.

**Table 1:** Reference values

	**Normal value**	
**ESR**		[14]
**Women ≤ 50 years of age**	<20 mm/hour	
**Women > 50**	<30 mm/hour	
**Men ≤ 50 years of age**	<15 mm/hour	
**Men > 50**	<20 mm/hour	
**Haemoglobin**		[14]
**Women**	≥ 7.5 mmol/l	
**Men**	≥ 8.0 mmol/l	
**Gamma-glutamyl transferase**		[14]
**Women**	<35 U/l	
**Men**	<40 U/l	
**Cholesterol (97,5 percentile)**		[14]
**Women 20–29 years of age**	≤ 6.7 mmol/l	
**Women 30-39**	≤ 6.9 mmol/l	
**Women 40-69**	≤ 7.7 mmol/l	
**Men 20-29**	≤ 6.7 mmol/l	
**Men 30-39**	≤ 7.4 mmol/l	
**Men 40-69**	≤ 7.8 mmol/l	
**HDL cholesterol**		[14]
**Women**	<1.1 mmol/l	
**Men**	<0.9 mmol/l	
**Cholesterol/HDL ratio**		[15]
**No elevated risk**	<5	
**Elevated risk**	≥5	
**LDL cholesterol**	<2.5 mmol/l	[15]
**Triglycerids**	≤2.2 mmol/l	[14]
**Fasting blood glucose**	≤5.6 mmol/l	[14]
**Urinary protein**	negative	
**Urinary glucose**	negative	
**BMI**	<25	[16]
**Waist circumference**		[17]
**Women**	<80 cm	
**Men**	<94 cm	
**Systolic blood pressure**	≤140 mm Hg	[15]
**Diastolic blood pressure**	≤90 mm Hg	[18]

As indicated above, where applicable, values from the health declaration and medical examination were dichotomised into normal and abnormal values, and used as such in the analyses. Whether or not values from the health declaration and the medical examination were considered abnormal was based on generally accepted reference values taking age and gender into account, derived from WHO guidelines, GP guidelines and laboratory textbooks (see Table 1).

### Statistical analysis

Applicants who did not undergo a medical examination were excluded from the analyses. These participants were compared to the participants who had a general medical examination performed on gender, occupation and subsequent sickness absence using chi-square tests. Multivariable logistic regression with backward elimination was performed to assess which of the variables from the health declaration form and medical examination predicted sickness absence. For dichotomous variables only those that varied in the population studied (frequency of both categories ≥ 1%) were included in analyses. All variables mentioned in Table 2 were included in univariate analysis and factors significantly associated with the likelihood of subsequent sickness absence (P < 0.05) were included in the final model.

**Table 2:** Characteristics of the study population and potential predictors (n = 634)

			**Experienced sickness absence**		**Did not experience sickness absence**	
**Age (years) mean (SD) at application**	**35.5**	**(5.99)**	**35.2**	**(6.76)**	**35.5**	**(5.85)**
	**n (%)**		**n (%)**		**n (%)**	
**Sex**						
**Male**	418 (65.9)		41 (9.8)		377 (90.2)	
**Female**	216 (34.1)		50 (23.1)		166 (76.9)	
**Occupations**						
**Other medical doctors/specialists**	154 (24.3)		21 (23.1)		133 (24.5))	
**Legal professions**	130 (20.5)		11 (12.1)		119 (21.9)	
**General practitioners**	130 (20.5)		12 (13.2))		118 (21.7)	
**Dentists/orthodontists**	84 (13.2)		17 (18.7)		67 (12.3)	
**Paramedic professions**^**1**^	33 (5.2)		8 (8.8)		25 (4.6)	
**Technical professions**^**2**^	39 (6.2)		4 (4.4)		35 (6.4)	
**Financial services3**	29 (4.6)		5 (5.5)		24 (4.4)	
**Pharmacists**	17 (2.7)		2 (2.2)		15 (2.8)	
**Veterinarians**	11 (1.7)		5 (5.5)		6 (1.1)	
**Midwives**	7 (1.1)		6 (6.6)		1 (0.2)	
**Total**	634 (100)		91 (100)		543 (100)	
**Sports (yes)**	463 (87)		66 (12.4)		397 (74.6)	
**Smoking (no)**	549 (86.7)		75 (11.8)		474 (74.9)	
**Alcohol (≤2 units/day)**	607 (96.3)		89 (14.1)		518 (82.2)	
**Fam.hist heart disease (no)**	523 (93.1)		70 (12.5)		453 (80.6)	
**Self reported working hours (≤50)**	375 (72.7)		54 (10.5)		321 (62.2)	
**Health complaints (yes)**	375 (59.1)		63 (10)		312 (49.2)	
**Present medication use (no)**	510 (80.6)		69 (10.9)		441 (69.7)	
**Additional examination (no)**	467 (73.8)		63 (10)		404 (63.8)	
**Prior sickness absence (no)**	534 (84.6)		68 (10.6)		466 (73.9)	
**BMI (<25)**	425 (67.7)		65 (10.3)		362 (57.4)	
**Waist circumference (normal)**	415 (68.6)		49 (8.1)		366 (60.5)	
**Consultation of GP (yes)**	544 (86.5)		78 (12.4)		466 (74.1)	
**Consultation physiotherapist (no)**	396 (63)		45 (7.2)		351 (55.8)	
**GGT (normal)**	529 (93.5)		70 (12.4)		459 (81.1)	
**ESR (normal)**	560 (96.6)		75 (12.9)		485 (83.6)	
**Chol/HDL (normal risk)**	509 (84.3)		73 (12.1)		436 (72.2)	
**Triglycerides (normal)**	510 (93.9)		69 (12.7)		441 (81.2)	
**Blood glucose (normal)**	532 (87.2)		74 (12.1)		458 (75.1)	
**Hb (normal)**	547 (97)		72 (12.8)		475 (84.2)	
**Urinary analysis (no abnormalities)**	563 (97.1)		80 (13.8)		483 (83.3)	

^1^Paramedic professions: physiotherapists, chiropractors, psychologists, dental hygienists, podotherapists, speech therapists.

^2^Technical professions: trainers, interim-managers, marketing managers, ICT professionals, mediators, engineers.

^3^Financial services: accountants, tax advisors, insurance agents.

As the aim of this study was to find the best set of predictors for future periods of sickness absence confounding of certain variables was not formally addressed. Naturally, the associations between independent and the dependent variable in the multivariable models, were mutually adjusted.

As there was a considerable number of missing values, especially for the data from the medical examination, it was decided to impute missing data for these variables using chained imputations [19] with an imputation model consisting of all the potential predictors as well as the dependent variable. Trace plots of means and standard deviations of imputed variables were checked for convergence. After convergence had been observed from the trace plots, Rubin’s rules were applied to derive regression coefficients for the potential predictors. In this process, it was also examined whether the number of imputations influenced the results. It was found that results were stable after 50 imputations, which is what was used in the final analyses. In addition, complete case analyses were compared with the results from the imputed datasets to examine whether unexpected or extreme differences occurred.

In addition we examined whether the associations differed between males and females by including interactions with gender to the univariable as well as to the final model. Interactions were not examined in the full model because this would have led to too many independent variables in that model. Interactions were deemed statistically significant at an alpha of 0.10. No stratification based on age was performed because the study population did not show much variation with regard to age.

Finally, three post hoc analyses were performed. First, to investigate the influence of pregnancy-related sickness absence on gender differences in sickness absence, a separate multivariable regression analysis was run in which cases with pregnancy related periods of sickness absence were excluded. Second, to investigate whether the association between gender and sickness absence was influenced by the fact that the midwives exclusively consisted of women, these midwives were excluded from the multivariable analysis. Lastly, a multivariable regression analysis excluding the applicants pregnant at the time of application was performed to evaluate the influence of possibly pregnancy-induced abnormalities in the blood tests. An alpha of 0.05 was used to indicate statistical significance for all analyses and all of these were conducted in STATA version 12.1.

## Results

### Participant characteristics

The group accepted for insurance cover in 2003 and insured during the full follow-up period consisted of 819 persons. Of these, 634 applicants (77.4%) underwent a medical examination and were included in the analyses. The chi-square tests comparing those who underwent a medical examination with those who were accepted for insurance cover without was significant for gender, profession and the outcome variable (subsequent periods of disability). More men than women underwent a medical examination (p < 0.001) and those who did not undergo a medical examination more often experienced a subsequent episode of sickness absence (p = 0.028). The different professions were unevenly distributed in these two groups as well; more medical doctors/specialists underwent an examination and fewer general practitioners and dentists/orthodontists (p < 0.001).

Of the 634 applicants results from the medical examination were incomplete for 249 with one or more variables missing. Table 2 presents percentages for demographic variables (sex, age, profession) together with the other potential predictors of sickness absence.

### Predictors of sickness absence

All variables showing a frequency of both categories >1% are listed in Table 2 and were assessed as potential predictors of sickness absence. Table 3 shows the results from the multivariable logistic regression analyses. Female gender (OR 2.04, 95% CI 1.23-3.38, p = 0.006), prior periods of sickness absence (2.07, 95% CI 1.15-3.76, p = 0.016), any health complaints listed in the health declaration (OR 1.88, 95% CI 1.10-3.20, p = 0.02) and elevated ESR (OR 4.05, 95% CI 1.54-10.64, p = 0.004), raised the odds of subsequent periods of sickness absence. The nature of the occupation also proved to be related to the outcome variable in a statistically significant way (OR ranging from 1.22 for GPs to 56.61 for midwives compared to legal professionals).

**Table 3:** Variables related to sickness absence in follow-up from the multivariable logististic regression model

	**OR univ**	**p**	**95% CI**	**OR multiv**	**p**	**95% CI**
**Gender**	2.77	0.730	1.76-4.35	2.04	0.006	1.23-3.38
**Profession**						
**Legal professions**	1			1		
**General practitioners**	1.10	0.827	0.47-2.59	1.22	0.660	0.50-2.99
**Technical professions**	1.24	0.730	0.374.12	1.38	0.620	0.39-4.90
**Pharmacists**	1.44	0.654	0.29-7.14	1.43	0.678	0.27-7.62
**Other medical doctors/specialists**	1.71	0.173	0.79-3.69	1.98	0.095	1.49-4.43
**Financial services**	2.25	0.164	0.72-7.08	3.12	0.061	0.95-10.31
**Dentists/orthodontists**	2.74	0.015	1.21-6.20	3.28	0.007	1.37-7.81
**Paramedic professions**	3.46	0.016	1.26-9.48	4.71	0.004	1.65-13.46
**Veterinary surgeons**	9.02	0.001	2.37-34.36	12.61	0.001	3.00-53.04
**Midwives**	64.91	0.000	7.15-588.90	56.61	<0.001	5.88-544.97
**Previous sickness absence periods**	2.11	0.006	1.24-3.60	2.07	0.016	1.15-3.76
**Health complaints**	1.71	0.028	1.06-2.78	1.88	0.02	1.10-3.20
**ESR**	4.04	0.003	1.63-10.04	4.05	0.004	1.54-10.64
**Year of birth <1963**	1					
**Year of birth 1963-1973**	0.61	0.089	0.35-1.08			
**Year of birth >1973**	1.34	0.929	0.54-1.96			
**Sports**	1.15	0.712	0.55-2.40			
**Smoking**	1.49	0.191	0.82-2.70			
**Alcohol (≤2 units/day)**	0.55	0.419	0.13-2.37			
**Fam.hist heart disease**	1.63	0.082	0.54-2.84			
**Self reported working hours (≤50)**	0.95	0.851	0.54-1.67			
**Present medication use**	1.39	0.218	0.82-2.36			
**Additional examination**	1.23	0.405	0.75-2.01			
**BMI (<25)**	0.81	0.407	0.50-1.33			
**Waist circumference**	1.84	0.880	0.48-1.62			
**Consultation of GP**	1.33	0.717	0.58-2.23			
**Consultation physiotherapist**	1.81	0.010	1.16-2.85			
**GGT**	1.30	0.574	0.52-3.20			
**Chol/HDL**	0.79	0.491	0.40-1.55			
**Triglycerides**	0.66	0.489	0.20-2.17			
**Blood glucose**	1.19	0.614	0.61-2.30			
**Hb**	1.51	0.510	0.44-5.14			
**Urinary analysis**	1.78	0.313	0.58-5.46			

OR univ = odds ratio in univariable analysis.

OR multiv = odds ratio in final multivariable model.

In the examination of interactions of gender with the potential predictors the only statistically significant interaction we observed was for one of the dummies of profession with gender. In males GP’s had a 3.26 times higher odds of sickness absence compared to legal professionals (95% CI: 0.89 to 11.94), whereas the OR for females was 0.40 (95% CI: 0.10 to 1.66). Generally the complete case analysis showed similar results to the results based on the imputed datasets. Results from the first post hoc sensitivity analysis showed that there was a changed relation between gender and sickness absence (OR 1.64, 95% CI 0.97 -2.77; p = 0.064) after having excluded women with pregnancy-related sickness absence from the analyses, with gender losing its statistical significance. The second post hoc sensitivity analysis in which midwives were excluded led to an unchanged result (OR 2.45, 95%CI 1.51-3.98; p < 0.001). Finally, the third post hoc analysis excluding 9 applicants pregnant at application showed a relation between elevated ESR and subsequent sickness absence which was less strong and no longer significant (OR = 2.62, 95%CI: 0.79-8.73; p = 0.116).

## Discussion

Predisposing risk factors for sickness absence periods of 30 days or more in a group of college and university educated self-employed were female gender, prior periods of sickness absence, health complaints listed in the health declaration and elevated ESR. Moreover, our results showed that the nature of the occupation was associated with the outcome variable in a statistically significant way with veterinarians and midwives having the highest odds and legal and technical professions the lowest odds of subsequent sickness absence. Our analysis did not show any substantial evidence for a difference in risk profile between males and females. The variables studied were considered to be of predictive value regarding subsequent sickness absence by insurance companies but had never been evaluated. Our results show that variables derived from the medical examination are of limited value in predicting sickness absence and disability.

In our study women had significantly raised odds to experience periods of sickness absence. These findings are consistent with previous studies in populations of employees [20-23] and with a study in a population applying for insurance [12]. The separate regression analysis in which pregnancy related periods of sickness absence were excluded demonstrates that the effects of gender for a large part must be attributed to reproductive complications. Several authors have also proposed a difference in working conditions to possibly account for gender differences in sickness absence [24,25]. However, this does not explain the increased risk of women because both gender and occupation were in the final model, and as such mutually adjusted. In addition, a separate multivariable regression analysis with the midwives, the only exclusively female profession excluded still showed gender to be significantly related to the outcome variable so it is unlikely that the effect we found is caused by working conditions for midwives only. Although gender specific differences in working conditions within professions cannot be ruled out it is improbable that this wholly explains the difference found.

The finding that previous periods of sickness absence raise the odds of experiencing subsequent periods is in agreement with research in groups of employees [26-30]. It cannot be derived from our study whether this is the result of pre-existing vulnerability, or of specific diseases or circumstances that tend to recur. It is, however, clear that self-employed with a history of previous sickness absence deserve additional attention.

One or more health complaints listed in the health declaration at application was associated with significantly higher odds of subsequent sickness absence. This association is well known for musculoskeletal disorders both in self-employed and employees [13,31,32] and for other aspects of medical history [12].

The nature of the occupation proved to be a strong predictor of sickness absence. Differences in sickness absence patterns between occupational classes and between groups with different socioeconomic status are well known from previous research in employees [33,34]. As stated previously, our study population consisted of a homogenous group considering socioeconomic status. Data on specific working conditions was unavailable but physical and mental job characteristics are known to differ between occupations and these differences are relevant with regard to the risk of sickness absence in populations of employees [35,36]. Those in paramedic professions and dentists/orthodontists had a considerably higher risk of sickness absence than general practitioners and those in legal professions. This may be reflective of more physically demanding work characteristics. Midwives and veterinary surgeons experienced the highest risk of sickness absence. Although these groups were limited in size, and our sample in these groups was probably biased towards less healthy persons, it is clear that there are differences in occupational risk between the professional categories in our study. Further studies, which take the different working conditions into account, will need to be undertaken. Furthermore, it cannot be ruled out that these occupations attract self-employed with characteristics that are related to vulnerability to sickness absence.

The association of erythrocyte sedimentation rate (ESR) and subsequent sickness absence was unanticipated. Use of the ESR as a screening test in asymptomatic persons is generally not recommended because of low sensitivity and specificity. Therefore, the association found comes as a surprise and cannot be adequately explained. Only twenty of the applicants had an elevated ESR and of these, 6 applicants were pregnant, which is known to raise ESR [14] and one had an inflammatory disease. On leaving the pregnant applicants out of the analysis, the elevated ESR lost its statistical significance (OR = 2.62, 95%CI: 0.79-8.73; p = 0.116); therefore pregnancy can be assumed to at least partly explain the relation found.

Life style factors, such as smoking, heavy consumption of alcohol and lack of physical activity as reported by the insurance applicants, were not associated with subsequent sickness absence, although these associations are well established in studies on employees [21,37,38]. A possible explanation is that the more extreme unhealthy behaviours were rare in our population: 12.4% were current smokers and only 3.7% reported drinking 3 or more units of alcohol per day. As data from self-report was used for these variables, underreporting may also have been of influence. Also unexpectedly, no effect was found of the BMI on future periods of sickness absence. A considerable amount of literature has been published on the relation of a high relative weight and periods of sickness absence in employees [21,39,40]. Our finding is, however, in agreement with Hamilton’s study on insurance applicants [12]. Obesity may have been too infrequent in this sample to find an effect, as only 3.5% of applicants had a BMI over 30, or the follow-up period may have been too short because the negative health effects of obesity take some time to develop. Also surprisingly age at application did not contribute to predicting sickness absence. This may be explained by the lack in variation in age in our study population (mean age 35.5 years old, SD 5.99).

Predictive variables, identifying individuals at risk of experiencing sickness absence can also be of value as these give an improved opportunity for prevention. Interventions to prevent sickness absence in self-employed are largely uncharted territory. Our study findings can be of value to target the self-employed that are most at risk for sickness absence thus contributing to effectively supporting this economically important group.

### Strengths and limitations

One of the strengths of our study was the use of files from an insurance company, providing us with reliable data on self-employed, a population that is otherwise difficult to study. Although variables predictive of sickness absence have been studied in groups of employees this has never been investigated in a population of self-employed until now. Additionally the study relies on factual data collected at application for an insurance policy and registered data on periods of sickness absence from the insurance company, which prevented recall-bias. Another strength of our study is the long follow-up period of almost 8 years. Also, the nature of the dataset allowed us to study predisposing variables and thus to evaluate the widespread practice in the insurance business of emphasizing strictly medical variables as predictors of subsequent sickness absence. By limiting our definition of the outcome variable to periods of sickness absence of 30 days or more, minor ailments were excluded. In our opinion this provides a more solid base to our findings in predicting especially long-term sickness absence.

An important limitation derives from the sample size. Although the study population was reasonably large it consisted of young self-employed and the number of applicants with abnormalities at the medical examination was in general small. In addition, for some of the questions in the health declaration form there was hardly any variation in answers given. This means that the power to detect relations between these factors and sickness absence was limited. Other limitations are related to the selection of our sample used for analysis. First almost 22% of the sample did not undergo a medical examination and were therefore not included in the analyses. However, whether or not an applicant underwent a general medical examination was not related to health characteristics, but to the sum insured only. Nonetheless these applicants more often experienced a subsequent episode of sickness absence. This can largely be explained by the fact that the group that did not undergo a medical examination comprised more women. When odds ratios were calculated for men in relation to women the effect of gender on subsequent sickness absence was 1.5 times higher for those who did not undergo an examination. This intimates an underestimation of the effect of gender on subsequent sickness absence in our study. Secondly, although the missing variables were probably missing-at-random as for all applicants the same examination was requested, it cannot be ruled out that this has somewhat influenced relations in our multivariable model. We did, however, impute missing values based on a large number of other variables. With regard to selection there was on one hand possibly an overrepresentation of less healthy persons in our study population. As disability insurance operates in a competitive market the insured sometimes move to a competing insurance company that offers more favourable premiums. Only those in good health can easily change insurance company. As we only included applicants with a full follow-up period this may have inflated the odds for experiencing a period of sickness absence in our study for these two occupations. Also persons tend to take out insurance when they anticipate a higher risk of sickness absence, thus possibly further elevating the risk of sickness absence in our population. Lastly our study consisted of applicants accepted for insurance cover only, excluding those who were denied an insurance contract, possibly because of very serious health problems. As legislation in the Netherlands forces insurance companies to destroy records of those who are refused insurance cover, the extent of this issue is not known. This last issue may bias our selection towards applicants in better health. All issues regarding selection bias taken into consideration, this probably means that our sample approximates the health state of the population of higher educated self-employed and that the risk estimates from our study are not heavily biased from these forms of selection.

A final limitation relates to whether our study results are generalizable. Our study population consisted of well educated self-employed with a private disability insurance policy only. Some caution must therefore be applied as to whether our findings are transferable to other populations. Highly educated are, however, an economically important and growing group of self-employed [41] and therefore deserve attention in their own right.

## Conclusions

Our study focussed on predisposing factors for periods of sickness absence of 30 days or more in self-employed. The risk factors found in our study show overlap with variables found in groups of employees but not all results from these studies could be replicated. Sickness absence was found to be related to demographic variables (gender, profession), medical variables (health complaints and ESR) and to variables with both a medical and a behavioural component (previous sickness absence). These results imply that college and university educated women in self-employment are vulnerable to sickness absence. Self-employed with a history of previous sickness absence and those in high-risk professions deserve additional attention as well as they too have an increased risk of sickness absence. The emphasis put on results from the general examination by insurance companies seems unjustified.

## Competing interests

ECW works at Movir, the insurance company that provided the data. No additional funding was received and Movir had no involvement regarding analysis and interpretation of the data, writing the manuscript or in the decision to submit the paper for publication.

## Authors’ contributions

ECW: was involved in the conception and design of the study, carried out the data collection, was involved in the statistical analysis and drafted the manuscript. SB: was involved in the conception and design of the study, and assisted to draft the manuscript. MRdB: conducted the statistical analysis and assisted to draft the manuscript. JJLvdK: coordinated the study and delivered improvements to the manuscript. All authors have approved the final manuscript and agree with its submission to BMC Public Health. All authors read and approved the final manuscript.

## Pre-publication history

The pre-publication history for this paper can be accessed here:

http://www.biomedcentral.com/1471-2458/14/420/prepub

## Supplementary Material

Additional file 1 **Disability insurance schemes for self-employed in the Netherlands.** In contrast to workers with an employer, self-employed are not covered by public disability insurance systems. Insurance against the risk of long-term incapacity for work has been left to the private insurance market and is voluntary. The self-employed can choose between different companies, can choose the amount they want to insure and a deferment period, i.e. the waiting period before the insurance company starts paying benefits. The insurance company is allowed to assess risks at the start of the insurance contract. Risk assessment for disability insurance is based on a filled out health declaration form and, depending on the sum insured, a general medical examination. The decision whether or not to request a general medical examination is unrelated to the health of the applicant, only to the sum insured. In case of specific health problems or risk factors medical information from treating physicians can be requested or a specific examination targeted at the health risk can be performed [11]. The insurer cannot end the insurance policy in case of an unfavourable claims history or other health-related issues, only the insured can. Reasons to do this can be change of occupational situation (and therefore no longer any need for private disability insurance) or more favourable terms of insurance with another company. Click here for file
