# Supplementary material for: Predictors of sickness absence in college and university educated self-employed: a historic register study
Source: BMC Public Health. 2014 May 2;14:420. doi: 10.1186/1471-2458-14-420 (PMC4108014; doi:10.1186/1471-2458-14-420)
Supplement: Additional file 1 — Disability insurance schemes for self-employed in the Netherlands. In contrast to workers with an employer, self-employed are not covered by public disability insurance systems. Insurance against the risk of long-term incapacity for work has been left to the private insurance market and is voluntary. The self-employed can choose between different companies, can choose the amount they want to insure and a deferment period, i.e. the waiting period before the insurance company starts paying benefits. The insurance company is allowed to assess risks at the start of the insurance contract. Risk assessment for disability insurance is based on a filled out health declaration form and, depending on the sum insured, a general medical examination. The decision whether or not to request a general medical examination is unrelated to the health of the applicant, only to the sum insured. In case of specific health problems or risk factors medical information from treating physicians can be requested or a specific examination targeted at the health risk can be performed [11]. The insurer cannot end the insurance policy in case of an unfavourable claims history or other health-related issues, only the insured can. Reasons to do this can be change of occupational situation (and therefore no longer any need for private disability insurance) or more favourable terms of insurance with another company. [file 1471-2458-14-420-S1.docx]

Additional file 1

Disability insurance schemes for self-employed in the Netherlands

In contrast to workers with an employer, self-employed are not covered by public disability insurance systems. Insurance against the risk of long-term incapacity for work has been left to the private insurance market and is voluntary

The self-employed can choose between different companies, can choose the amount they want to insure and a deferment period, i.e. the waiting period before the insurance company starts paying benefits.

The insurance company is allowed to assess risks at the start of the insurance contract. Risk assessment for disability insurance is based on a filled out health declaration form and, depending on the sum insured, a general medical examination. The decision whether or not to request a general medical examination is unrelated to the health of the applicant, only to the sum insured. In case of specific health problems or risk factors medical information from treating physicians can be requested or a specific examination targeted at the health risk can be performed. [11]

The insurer cannot end the insurance policy in case of an unfavourable claims history or other health-related issues, only the insured can. Reasons to do this can be change of occupational situation (and therefore no longer any need for private disability insurance) or more favourable terms of insurance with another company.
